# KI bei der Operationsplanung adulter spinaler Deformitäten: Analyse eines KI-basierten Werkzeuges zur Korrekturplanung und -umsetzung mithilfe patientenindividueller Stäbe

**DOI:** 10.1007/s00132-026-04831-y

**Published:** 2026-05-04

**Authors:** Patrick Strube, Alexander Hölzl, Anna-Maria Vogel, Georg Matziolis, Chris Lindemann

**Affiliations:** Orthopädie, Campus Waldkliniken Eisenberg, Universitätsklinikum Jena/Friedrich Schiller Universität Jena, 07607 Eisenberg, Deutschland

**Keywords:** Künstliche Intelligenz, Osteotomie, Planungstechnik, Skoliose, Wirbelsäule, Artificial intelligence, Osteotomy, Planning technic, Scoliosis, Spine

## Abstract

**Hintergrund:**

Eine optimale Alignment-Korrektur hat das Potenzial, die hohen Komplikationsraten von Operationen adulter Skoliosen zu verbessern. KI-Werkzeuge versprechen dabei, Analyse und Planung zu erleichtern und über patientenindividuelle Anfertigung von Stäben die operative Umsetzung zu verbessern.

**Ziel der Arbeit:**

Die Untersuchung eines KI-basierten Werkzeuges hierfür.

**Material und Methoden:**

Analyse der ersten 10 Fälle adulter Skoliosen unter Nutzung des KI-Werkzeuges in unserer Klinik hinsichtlich Ergebnissen und Limitationen.

**Ergebnisse:**

Die Methode ermöglicht eine zeitsparende Analyse und eine signifikante Verbesserung des Wirbelsäulenprofils. Limitationen resultieren aus unberücksichtigten Parametern im Modell und durch willkürliches Bias durch den Planer, die Einfluss auf die Operationserfolgsrate adulter Skoliosen haben.

**Diskussion:**

Das vorgestellte KI-Werkzeug stellt eine praktikable und einfach anzuwendende Methode dar, die Balance von Patienten wiederherzustellen. Die Erweiterung des Modells könnte die Komplikationsraten in Zukunft senken.

## Abstract (en)

**Background:**

Optimal alignment correction can potentially lower the high complication rates of adult scoliosis surgeries. AI tools can help make the analysis and planning process easier as well as improve the surgical realization via patient-specific rod manufacturing.

**Objectives:**

Investigation of an AI-based tool regarding this.

**Materials and methods:**

Analysis of the first 10 AI-tool used-cases of adult scoliosis in our department regarding outcome and limitations.

**Results:**

The method allows time-sparing analysis and significant improvement of the spine profile. Limitations result from unimplemented parameters in the model and from arbitrary bias caused by the planner, which influence the surgical success rate of adult scoliosis.

**Conclusions:**

The presented AI-tool represents a practicable and easy to use method for restoring the balance of patients. Extension of the model could potentially lower complication rates in the future.

In den letzten beiden Dekaden ist das Wissen um das wahrscheinlich ideale individuelle spinopelvine Alignment stetig gewachsen. Dennoch sind die Planung und Umsetzung desselben im Rahmen der Korrektur adulter spinaler Deformitäten aufgrund multipler Faktoren schwierig. Im Rahmen dieses Artikels soll ein KI-basierter Ansatz vorgestellt und kritisch evaluiert werden, der diese Problematik adressiert.

## Hintergrund

Adulte Wirbelsäulendeformitäten lassen sich in unterschiedliche Ursachen unterteilen, wie de-novo degenerative Prozesse, degenerativ dekompensierte idiopathische Skoliosen und Hyperkyphosen oder Deformitäten sekundärer Ursachen, die vielfältiger Natur sein können [1]. Dennoch ist all diesen Formen gemeinsam, dass sie mit zunehmender Deformität und Dysbalance auch zu einer zunehmenden Einschränkung der Lebensqualität führen. Diese ist über alle adulten Deformitäten betrachtet etwa so groß, wie die bei einem Diabetes mellitus oder bei einer Krebserkrankung. Bei einer sagittalen Imbalance von mehr als 10 cm gibt es sogar keine in der Literatur vergleichbare Erkrankung hinsichtlich der Ausprägung der Lebensqualitätseinschränkung [4]. Die Behandlung der Patienten mit adulten Skoliosen ist komplex und umfasst sowohl konservative, semiinvasive als auch operative Therapieoptionen. Die operative Therapie wiederum ist nach Versagen der konservativen und semiinvasiven Maßnahmen und bei entsprechendem Leidensdruck oder einer Dekompensation mit einhergehenden akuten neurologischen Defiziten indiziert. Das Operationsspektrum ist äußerst breit und umfasst nahezu alle möglichen wirbelsäulenchirurgischen Optionen von alleiniger Dekompression bis hin zur Korrektur der Deformität mit gleichzeitigem Adressieren eventuell bestehender neuraler Stenosen, wobei man im Rahmen des Entscheidungsprozesses patientenindividuell sowohl den Entschluss zur Operation an sich als auch das Ausmaß des zumutbaren Eingriffs der häufig komorbiden Patienten abwägen muss. Im Rahmen dieser Operationsentscheidung befinden wir uns jedoch immer noch in einem Spannungsfeld bezüglich der Erfolgsraten der operativen Behandlung und den hohen Komplikationsraten derselben. Schon 2009 konnten Bridwell et al. zeigen, dass die Lebensqualität bei den Patienten unter nichtoperativer Therapie eher sinkt, während die operative Behandlung zu einer signifikanten Steigerung der Lebensqualität führt [6]. Dennoch erreicht dieser Effekt nicht alle Patienten gleichermaßen. Nur ein Drittel der Patienten kehrt bei Korrektur auf Normalwerte der Durchschnittbevölkerung bezüglich des Schmerzes und der Aktivität zurück, etwa ein Viertel profitiert nicht. Den größten Benefit haben Patienten mit präoperativ moderater bis schwerer Einschränkung, wobei es für letztere nahezu unmöglich ist, das Niveau der Normalbevölkerung zu erreichen [28]. So kann man zwar feststellen, dass die Operation die Lebensqualität besser als die konservative Behandlung steigert, jedoch zu welchem Preis? Die Komplikationsrate von Korrekturoperationen lag vor etwa 15 Jahren bei ca. 70 % 2 Jahre nach der Operation [34]. Im Rahmen des Wissenszuwachses bezüglich sagittalem Alignment und der Verbesserung der operativen Technik sind 2021 Komplikationsraten von „nur noch“ 58 % publiziert worden [18]. In einer kürzlich veröffentlichten Survivalanalyse von über 1000 Patienten hatten über 50 % eine Komplikation innerhalb der ersten 38 Tage nach der Operation, nur ein Drittel hatte ein komplikationsfreies 2‑Jahres-Intervall, mehr als 10 % mussten innerhalb 52 Tagen nach Operation revidiert werden und die 2‑Jahres-Revisionsrate lag bei 23 % [17]. Hauptgrund für die hohen Komplikations- und Revisionsraten sind neben mechanischem Versagen der Implantate vor allem proximal junktionale Versagensmechanismen (PJF), wie kraniale Anschlusskyphosierungen, -frakturen, Implantatdislokationen etc. Um diesen entgegenzuwirken, wurden verschiedene Strategien verfolgt, wie die Nutzung von junktionalen Fixationstechniken mit Haken oder Bändern [7], Multistabkonstrukte [12], Nutzung von rhBMP‑2 zur Fusionsinduktion [2] sowie die Optimierung der Alignmentziele [23].

## Sagittale Alignmentkorrektur

Diese Alignmentziele wurden in der Literatur der letzten Jahrzehnte vielfältig untersucht. Es wurden dabei verschiedene Kriterien abgeleitet, die einerseits helfen, das Ausmaß und die Lokalisation der individuellen Deformität besser einschätzen zu können und andererseits eine Planung zur Wiederherstellung der individuell optimalen Wirbelsäulenkontur und Balance abzuleiten. Für die sagittale Imbalance hat sich der sogenannte Global Alignment and Proportion (GAP) Score etabliert [37], in dem die Kategorien Beckenkippung, Lordoseausprägung, Lordosedistribution, globale Balance und Alter einfließen. Basisparameter für die individuelle Kalkulation ist in der Regel die Beckendicke der Patienten in Form des Parameters „pelvic incidence“ (PI). Es konnte gezeigt werden, dass sowohl die operative Verbesserung des GAP-Scores als auch die Rekonstruktion des individuellen Wirbelsäulentyps nach Roussouly [31] zu einer Reduktion der mechanischen Versagensrate führen können [9, 16]. Dementsprechend erscheint es vielversprechend, diese Rekonstruktion möglichst genau durchzuführen, um die Komplikationsraten niedrig zu halten bzw. weiter zu senken.

### Korrekturplanung

Bisher war allein die Planung ein aufwändiger Prozess, in dem mehrere Winkel der Wirbelsäule manuell gemessen werden mussten, um die Deformität zu erfassen. Das Spektrum der Messungen hierbei ist immens. So misst man die erwähnte PI, den sakralen Slope, die Beckenkippung, die lumbale Lordose, die thorakale Kyphose, den „Femur-obliquity“-Winkel, den Abstand vom C7-Lot zur Sakrumhinterkante und in Relation zu den Hüftkopfzentren, den T1-Tilt, die koronare Lotverschiebung zur Sakrummitte, Schulter- und Beckenschiefstand und ggf. viele mehr. Hierauf basierend können anhand des GAP-Scores eine Analyse der sagittalen spinopelvinen Balance erfolgen und Behandlungsziele bezüglich des Orts und Ausmaßes der Korrektur festgelegt werden. Zur Korrekturplanung haben sowohl Arbeitsgruppen um Claudio Lamartina als auch um Jean Charles Le Huec Korrekturvorschläge erarbeitet, die jedoch ebenfalls auf den ersten Blick nicht trivial erscheinen [3, 21]. Sind nun die Maße erhoben und die Korrekturwinkel bestimmt, muss eine chirurgische Strategie festgelegt werden, mit der dieses geplante Ziel am wahrscheinlichsten erreicht werden kann. Hierzu benötigt man gegebenenfalls zusätzlich eine Analyse der Beweglichkeit der Wirbelsäulendeformität (z. B. in Form von Bending- und Funktionsaufnahmen), um das Ausmaß des knöchernen Releases vor Deformitätenkorrektur abschätzen zu können. Überlegungen betreffen hierbei Fragen wie: Reichen intersomatische Cages? Benötigt man zusätzlich Osteotomien? Welches Ausmaß müssen diese Osteotomien haben, z. B. Ponte-Osteotomien vs. 3-Säulen-Osteotomien?

### Korrekturausführung

Ist man zu einem guten Plan gekommen, gilt es nun, diesen umzusetzen. Für die Umsetzung stehen dem versierten Wirbelsäulenchirurgen mittlerweile viele moderne Werkzeuge, wie die intraoperative Navigation und Robotik zur Verfügung. Dazu kommen spezielle Meißel, Instrumente und Retraktoren für Osteotomien, distrahierbare Cages, sequenzielle Reducer sowie Doppelkopfschrauben und Seitkonnektoren für Doppelstabsysteme. Am Ende der Operation, nach suffizienter Dekompression und ausreichendem Release, steht dennoch die Korrektur basierend auf den manuell vom Chirurgen konturierten Stäben, wobei die Kontur dem Planungsziel entsprechen sollte. Die Fähigkeit, eine geplante Winkelkrümmung auf einen Stab zu übertragen, unterliegt jedoch leider einer erheblichen interindividuellen Varianz. So ließen Sardi et al. 2023 10 erfahrene Wirbelsäulenchirurgen Stäbe auf unterschiedliche Winkel biegen [32]. Die mittlere Winkelabweichung vom geplanten Zielwinkel beim Stabbiegen lag in dieser Studie bei immerhin 18,9°± 15,1°. Man darf hierbei nicht vergessen, dass die üblich verwendeten Stäbe in der Regel mindestens zwei Winkelbiegungen enthalten und in der Studie nicht geprüft wurde, wie genau die optimale Lokalisation der Winkel im Stabverlauf erreicht werden kann. So erscheint das Erreichen einer optimalen Stabbiegung durch den Chirurgen fast wie ein Glücksspiel und man könnte postulieren, dass die hohen mechanischen Komplikationsraten eine direkte Folge hiervon sind.

## Innovation des Prozesses durch KI

**Abb. 1:**
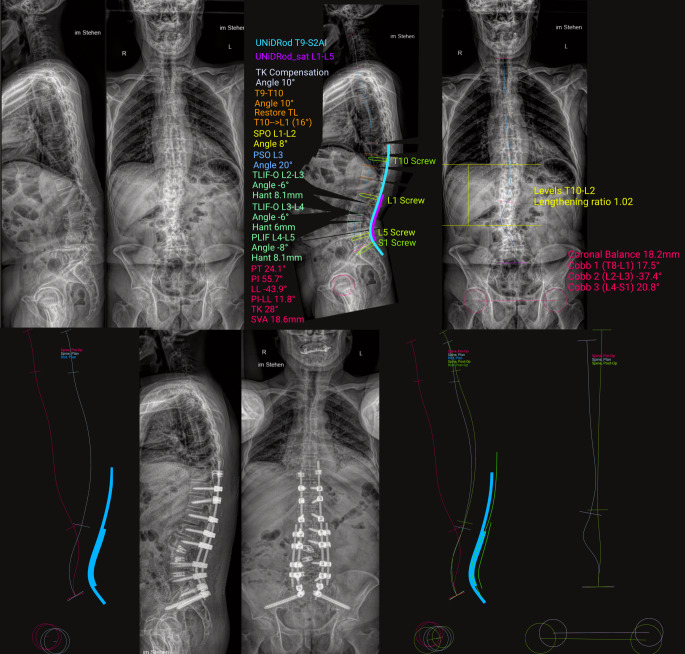
Die *obere Reihe* von Bildern zeigt (*links*) Wirbelsäulenganzaufnahmen (angefertigt mit EOS-Edge) von einem Patienten mit adulter de-novo Skoliose und konsekutiver ausgeprägter sagittaler und moderater koronarer Imbalance sowie (*rechts*) die KI-basierte Planungsskizze und Vermessung sagittaler und koronarer Parameter als Vorschlag der Versorgung nach Eingabe unserer Versorgungsstrategie (Osteotomien PSO und SPO, PLIF und TLIF in dargestellten Segmenten bei Versorgung Th10 bis S2Ala/Ilium mit Haken bei Th9, Doppelstabsystem). *Links* in der *unteren* *Reihe* ist schematisch das präoperative sagittale Profil *rot* und das virtuell simulierte Planprofil *weiß*, die konsekutive Stabkontur *hellblau* dargestellt. *Rechts* daneben sieht man die Versorgungsbilder am 5. postoperativen Tag (EOS Edge). In der *unteren Reihe* *rechts* erkennt man sagittal und koronar, wie die postoperative Kontur (*hellgrüne Linie*) der der Planung (*weiße Linie*) nahezu entspricht. *TK* thorakale Kyphose, *SPO* Smith-Peterson-Osteotomie, *PSO* Pedikelsubtraktionsosteotomie, *TLIF* transforaminale intersomatische lumbale Fusion, *PLIF* posteriore intersomatische lumbale Fusion, *Hant* Cage-Höhe anterior, *PT* „pelvic tilt“, *PI* „pelvic incidence“, *PI-LL* „pelvic incidence“ lumbale Lordose Mismatch, *SVA* sagittaler vertebraler Abstand

Seit geraumer Zeit sind zur Analyse der sagittalen Balanceparameter im Röntgenbild nun KI-basierte Werkzeuge vorhanden [25, 35]. Das diesbezüglich am weitesten entwickelte KI-Tool wird von einem Implantathersteller (UNiD^TM^, Medtronic, Dublin, Irland) zur Verfügung gestellt, wobei das Geschäftsmodell aus dem Verkauf der optimal konturierten Stäbe basierend auf der KI-Planung besteht. Das Werkzeug ermöglicht es, Wirbelsäulenganzaufnahmen pseudonymisiert in eine Plattform einzuspeisen. Der Chirurg determiniert nun im Planungsprozess die Strecke der Versorgung (Auswahl des oberen und unteren zu instrumentierenden Wirbels [UIV bzw. LIV]), die Lokalisation und die Art eventuell geplanter Osteotomien und die Lokalisation von intervertebralen Cages. Zusätzlich müssen Stabdicke und -material sowie deren Anzahl gewählt werden. Hiernach erfolgt die KI-Analyse des koronaren und sagittalen Profils, die Kalkulation der Zielwerte entsprechend den Idealwerten der Literatur (GAP-Score, Roussouly-Typ) sowie den Datensätzen, mit denen die KI trainiert wurde (siehe unten) und die Simulation des Ergebnisses anhand der Röntgenbilder inklusive der Adaptationsvorgänge in junktionalen nichtinstrumentierten Krümmungen. Die KI schlägt zum Teil mehrere Varianten der Versorgung vor, aus denen der Chirurg nach Erhalt der Planung in einem weiteren Schritt die Entscheidung trifft oder Modifikationen der Planung/Zielwerte vorschlägt. Aus dem Gesamtprozess entsteht am Ende in zwei bis drei kurzen Schritten eine dezidierte Operationsplanung inkl. der segmentalen Winkelkorrekturnotwendigkeit bei Cage-Versorgung oder Osteotomie sowie mit einer hieran angepassten, entsprechend der Literatur idealen, individualisierten Stabkonturierungsvorgabe. Abschließend kann man die Stäbe bestellen. Diese werden unsteril geliefert und können nach dem Sterilisationsprozess implantiert werden. Abb. 1 zeigt exemplarisch den ersten von uns mit KI-Werkzeug geplanten und operierten Fall samt Ergebnis.

Der Planungs- und Produktionsprozess dauert aktuell ca. eine Woche. Der Charme des Systems besteht darin, dass die Plattform anbietet, auch postoperative und Follow-up-Röntgenbilder einzuspeisen, um einerseits mittels Analysetools die Planung dem Ergebnis gegenüberzustellen und andererseits die KI-Planungsroutinen zugunsten guter Ergebnisse weiter zu optimieren, was nach Angabe des Anbieters stetig stattfindet. Somit resultieren mehrere Vorteile des neuen Werkzeuges:

1. Der Analyseprozess wird automatisiert und kostet erheblich weniger Zeit.
2. Der Planungsprozess wird automatisiert (ebenfalls Zeitersparnis) und ist wenig komplex.
3. Das Planungsergebnis wird vorab simuliert.
4. Eine manuelle Stabbiegung im Operationssaal entfällt.
5. Die Stäbe entsprechen der Planung.
6. Es besteht eine Kontrollmöglichkeit der Umsetzung des Plans.
7. Es ist ein selbstlernendes und sich verbesserndes System mit aktuell mehreren Zehntausend Datensätzen, die in die eigene Planung einfließen.

## Evaluation des KI-Werkzeugs

Seit November 2024 ist das genannte Werkzeug in den Waldkliniken Eisenberg zur Operationsplanung adulter Deformitäten im Einsatz.

### Patienten und Operationen

Von November bis März wurden damit 10 Patienten im Alter von 59–81 Jahren versorgt. Sechs Versorgungen waren von Th10-S2-Ala/Ilium, zwei von Th12-S2-Ala/Ilium und zwei L2-S2-Ala/Ilium. Drei Patienten hatten eine vorangegangene kurzstreckige Operation (mono- oder bisegmentale Fusion). In der Analyse der klinischen Parameter der untersuchten Kohorte bestand präoperativ eine deutliche funktionelle Einschränkung mit einem durchschnittlichen Oswestry Disability Index von 42,7 ± 10,3 %, was einer mäßig bis schweren Behinderung entspricht. Die gesundheitsbezogene Lebensqualität war entsprechend reduziert, mit einem EQ-5D-Indexwert von 0,54 ± 0,12. Die präoperativen Schmerzen wurden auf der numerischen Ratingskala (NRS) für Rückenschmerz mit 6,8 ± 1,2 und für Beinschmerz mit 5,2 ± 1,7 angegeben.

### Ergebnisse

Bereits am 5. postoperativen Tag zeigte sich eine deutliche Besserung des Beinschmerzes (NRS: 2,3 ± 1,4), während der Rückenschmerz mit 4,7 ± 1,3 noch moderat ausgeprägt war und teils der frischen Operationssituation zuzuschreiben ist.

**Abb. 2:**
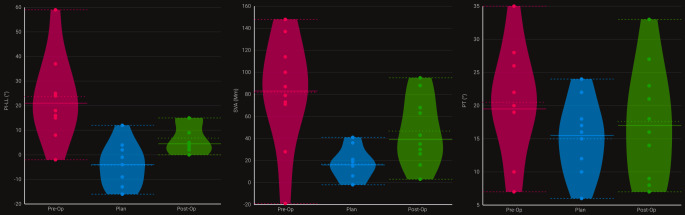
In der Abbildung erkennt man die Verteilung der präoperativen (*rot*), geplanten (*hellblau*) und postoperativen (*grün*) Parameter „pelvic incidence“-lumbale Lordose Mismatch (PI-LL, *links*), sagittaler vertebraler Abstand (SVA, Mitte) und „pelvic tilt“ (PT, *rechts*), analysiert von der KI-Plattform. Im Mittel (*horizontale Linien* in den Violinen) erkennt man zwar eine deutliche Verbesserung der Werte (von *rot* zu *grün*), jedoch wird hierbei der Plan (*hellblau*) meist nicht ganz erreicht. Die Verbesserung für PI-LL und SVA waren signifikant, nicht jedoch bei PT (Tab. 1), was darauf hindeutet, dass die postoperative Beckenrückkippung von der KI schwerer vorherzusagen ist, möglicherweise, weil eine verkürzte Hamstring-Muskulatur in manchen Patienten diese verhindert und dieser Muskelparameter nicht in der KI integriert ist

Die Analyse der radiologisch bestimmten sagittalen Parameter zeigte eine signifikante Verbesserung der sagittalen Ausrichtung im Vergleich zum präoperativen Zustand (Tab. 1). Insbesondere konnte durch die operative Korrektur eine Annäherung an die in der KI-gestützten Planung definierten Zielwerte erreicht werden. Dabei zeigten sich signifikante Unterschiede in mehreren Parametern, darunter die sagittale vertikale Achse (SVA, *p* < 0,001), die Lendenlordose (LL, *p* = 0,009) und die Differenz zwischen PI und LL (PI–LL, *p* < 0,001) (Tab. 1; Abb. 2).

**Tab. 1:** Vergleich sagittaler radiologischer Parameter zwischen präoperativ, Planung und postoperativ.

Radiologische Parameter	Präoperativ	Planung	Postoperativ	*P*-Wert
Sagittale vertikale Achse (SVA, mm)^*, ‡^	81,8 ± 49,5	18,5 ± 15,3	46,7 ± 30,7	< 0,001
Lumbale Lordose (LL, °)^*, †, ‡^	20,5 ± 19,6	49,9 ± 9,7	38,3 ± 7,8	0,009
Sakraler Slope (SS, °)^*^	23,6 ± 10,6	30,9 ± 9,2	27,3 ± 7,4	0,014
„Pelvic tilt“ (PT, °)	20,5 ± 8,1	16,4 ± 6,5	17,6 ± 8,6	0,104
„Pelvic incidence“–lumbale Lordose (PI-LL, °)^*, †, ‡^	23,7 ± 17,2	−2,3 ± 8,7	6,8 ± 6,2	< 0,001
T1 „pelvic angle“ (TPA, °)^*^	23,4 ± 8,5	13,1 ± 6,1	16,7 ± 5,8	0,001

Die Tabelle zeigt Mittelwerte ± Standardabweichung. *P*-Werte entstammen einer einfaktoriellen Varianzanalyse (1-way-ANOVA) mit Bonferroni-post-hoc-Test. Die Post-hoc-Testergebnisse sind folgendermaßen gekennzeichnet:

* *p* < 0,05 für präoperativ vs. Planung

† *p* < 0,05 für präoperativ vs. postoperativ

‡ *p* < 0,05 für Planung vs. postoperativ

Dennoch blieb in einigen Fällen eine Diskrepanz zwischen geplantem und erreichtem postoperativem Ergebnis bestehen, was auf patientenspezifische Faktoren wie segmentale Rigidität oder strukturelle Veränderungen zurückzuführen sein kann.

## Limitationen der Methode

Die dargestellten Fälle zeigen zwar einerseits das Potenzial des neuen KI-Werkzeugs, andererseits konnten wir aufgrund der geringen Zahl der Fälle bisher und des kurzen Follow-ups den klinischen Erfolg des Werkzeugs noch nicht darstellen. Ob sich durch dessen Anwendung tatsächlich eine Reduktion der mechanischen Versagensraten bzw. der proximal junktionalen Versagens- und Revisionsraten erreichen lässt, bleibt abzuwarten. Sicherlich ist der Lösungsansatz vielversprechend und bietet oben genannte Vorteile im Planungs- und Ausführungsprozess. Eine komplette Komplikationsvermeidung ist jedoch auch mit diesem Werkzeug aufgrund der im folgenden dargestellten Probleme (noch) nicht zu erwarten:

### Unzureichende Datenbasis und falsche Zielwerte

**Abb. 3:**
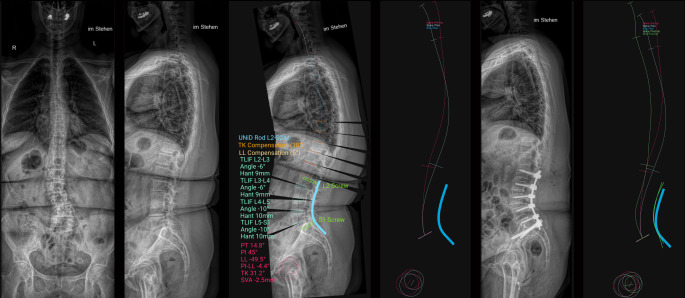
*Links* sieht man die präoperativen Wirbelsäulenganzaufnahmen einer Patientin mit adulter de-novo Skoliose und daneben die durch die KI-geplante Zielkorrektur mit Messwerten auf Basis der von uns vorgegebenen Versorgungsstrategie. Man erkennt in der Planung an den *schwarzen* Winkeln oberhalb L2, dass die KI eine harmonische Rekyphosierung der Brustwirbelsäule (BWS) auf sechs Segmente über der Versorgung verteilt errechnet hatte. Im Versorgungsbild *rechts* daneben erkennt man zwar eine suffiziente Korrektur in den versorgten Segmenten mit lumbaler Lordoserekonstruktion, die harmonische auf mehrere Segmente verteilte Rekyphosierung der BWS findet jedoch aufgrund des rigiden Thorax nicht statt. Stattdessen beobachtet man eine nahezu monosegmentale Kyphosierung im Segment L1/2. Die Diskrepanz wird auch im *rechten Bild* anhand der different verlaufenden *Linien* des geplanten (*weiß*) und postoperativen (*hellgrün*) thorakolumbalen Profils deutlich. Das Ergebnis kann eine vermehrte mechanische Lastverschiebung auf das kraniale Anschlusssegment verursachen und langfristig eine Anschlusssegmentdegeneration oder ein proximal junktionales Versagen begünstigen, anstatt beides – wie beabsichtigt – zu verhindern. *TK* thorakale Kyphose, *TLIF* transforaminale intersomatische lumbale Fusion, *Hant* Cage-Höhe anterior, *PT* „pelvic tilt“, *PI* „pelvic incidence“, *PI-LL* „pelvic incidence“ lumbale Lordose Mismatch, *SVA* sagittaler vertebraler Abstand

Die Literaturdatenbasis, anhand der das System Zielwerte definiert, haben nur einen positiven prädiktiven Wert von 76 % [20]. Viele Studien zeigen zudem, dass auch der GAP-Score für eine Vorhersage von Komplikationen allein unzureichend ist [10, 11, 27]. Somit wird man auch bei Erreichen der radiologischen Zielwerte Komplikationen erleben, die andere Ursachen haben. Zudem ist das Ausmaß der Korrektur selbst, unabhängig vom Erreichen der Zielwerte, ebenfalls ein unabhängiger Risikofaktor für ein Konstruktversagen [15, 26]. Es existieren mittlerweile gute altersadaptierte Zielwerte der Korrektur [19], die jedoch dem dargestellten Planungswerkzeug nicht automatisch zugrunde liegen. Eine manuelle Eingabe dieser ist jedoch im KI-Werkzeug als Zielvorgabe möglich und kann auf Dauer das System sicherlich verbessern. Osteoporose, speziell lokal in den Wirbeln am oberen Ende der Instrumentierung, ist ein weiterer wichtiger Risikofaktor für das Auftreten eines PJF [14, 36]. Dieser Faktor fließt jedoch in das bisherige Modell nicht ein. Muskelverkürzungen und ein rigider Thorax sind in der Patientenkohorte ebenfalls keine Seltenheit. Diese Parameter werden jedoch vom KI-Modell aktuell nicht berücksichtigt. Im Gegenteil simuliert das Modell in der Planung nicht selten kompensatorische Rekyphosierungen der Brustwirbelsäule, sodass eine harmonische Verteilung der Winkelanpassung suggeriert wird. Bei rigidem Thorax und verkürzter Rumpf- und Hamstring-Muskulatur sind jedoch dynamische multisegmentale Anpassungsvorgänge im Thoraxbereich und Hüftgelenk gegebenenfalls nicht möglich. Dementsprechend kann es, wie in Abb. 3 dargestellt, statt zu einer wie simuliert arkuären zu einer real angulären Kyphose oberhalb der Instrumentation kommen, was eine Anschlusssegmentdegeneration oder PJF begünstigt. Die schlechte Vorhersage des postoperativen PT durch die KI-Planung könnte ein Produkt unberücksichtigter Hamstring-Verkürzungen sein (Abb. 2 und Tab. 1), die eine Beckenrückkippung verhindern. Auch die fettige Infiltration der Rückenmuskulatur begünstigt das Auftreten einer mechanischen Komplikation [22]. Diese wird ähnlich der Osteoporose nicht erfasst, könnte jedoch in Zukunft in der MRT der Patienten mittels KI gemessen werden. Die Muskelverfettung korreliert mit dem Ausmaß der präoperativen sagittalen Imbalance und spielt so vielleicht eine bisher zu geringe Rolle in der Betrachtung der Problematik der PJF [24]. Passias et al. zeigten 2022, das ein hinsichtlich Frailty modifizierter GAP-Score (FAR-Score) eine deutlich höhere Korrelation mit der Komplikationsrate aufweist als der GAP-Score selbst [30]. Eine Erweiterung des KI-Modells um Frailty-Parameter wäre demnach ebenfalls sinnvoll und zielführend. Letzten Endes beruht die Datenanalyse immer auf statischen Bildern im Liegen oder Stehen, was dem natürlichen Bewegungsmuster der Patienten über den Tag nicht gerecht wird [33]. KI bietet die Möglichkeit, in Zukunft auch multiple Parameter von Bewegungsanalysen in die Planung der Versorgung mit einzubeziehen. Das wohl größte Problem ist jedoch aktuell, dass die Optimierungsstrategien dieses und vieler anderer Werkzeuge auf die Optimierung des radiologischen und nicht des klinischen Ergebnisses abzielen. Dies ließe sich leicht beheben, indem „patient reported outcome measures“ in das Modell integriert werden. Laut Anbieter ist eine solche Integration in Planung.

### Fehlende Strategieempfehlung

Eine weitere Limitation des Werkzeuges ist, dass der Chirurg selbst willkürlich bestimmte Parameter, die einen Einfluss auf die Komplikationsrate haben können, festlegen kann. Hierzu zählt allem voran die chirurgische Strategie (UIV, LIV, Cages, Osteotomien, junktionale Fixationstechniken, Stabmaterial, Stabdurchmesser etc.). So ist bekannt, dass es zu unterschiedlichen Raten von PJF kommen kann, je nachdem, ob der UIV z. B. bei L1 oder bei Th10 gewählt wird [8]. Han et al. zeigten daneben eine reduzierte Konstruktüberlebensrate bei Anwendung von Kobalt-Chrom-Stäben im Vergleich zu Titan-Stäben [13]. Eine KI-basierte Strategiefestlegung hinsichtlich des besten Outcomes wäre in der Zukunft wünschenswert.

### Stabkonturierung ist nicht Lordose

**Abb. 4:**
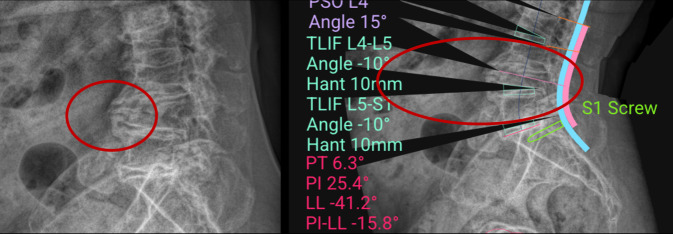
Grenzen der KI-basierten Planung bei ossär fixierten Segmenten. Im dargestellten Fall berechnet das KI-System eine großzügige lordosierende Korrektur durch ein anterior zu öffnendes Bandscheibensegment. Die Planung geht entsprechend der strategischen Vorgabe der TLIF von einer ausreichenden ventralen Mobilisierbarkeit des Segments aus, um gleichzeitig auch die gewünschte Lordose in diesem Segment zu erzielen (*rechtes Bild*, *rotes* *Oval*: ventral offener schwarzer Winkel bei L4/5, KI-Planung 10° segmentale Lordose mit ventraler Cage-Höhe – Hant von 10 mm). Im präoperativen Röntgenbild sind jedoch zirkumferente Osteophyten und die ossäre Fixierung des betroffenen Segments deutlich erkennbar (*roter Kreis* im *linken Bild*). Diese Befunde machen die errechnete Korrektur intraoperativ mit alleiniger TLIF nur unwahrscheinlich realisierbar. Dies verdeutlicht die Notwendigkeit einer kritischen Überprüfung der KI-Planung im Kontext struktureller Veränderungen, um Fehlschlüsse bei der Strategieauswahl und potenzielle postoperative Fehlstellungen zu vermeiden. *TLIF* transforaminale intersomatische lumbale Fusion, *PT* „pelvic tilt“, *PI* „pelvic incidence“, *PI-LL* „pelvic incidence“ lumbale Lordose Mismatch, *PSO* Pedikelsubtraktionsosteotomie

Die Konturierung des Stabes entsprechend dem chirurgischen Korrekturziel ist sicherlich wichtig. Dennoch erreicht man nicht immer die Lordose, die die KI-basierte Planung vorgibt. Maßgeblich für das Erreichen des Ziels ist auch das Ausmaß des knöchernen und Weichteil-Releases. Die KI berechnet, wie im dargestellten Fall (Abb. 4) großzügig, dass sich ein Bandscheibensegment anterior öffnen lässt, um Lordose zu generieren. Jedoch sind im präoperativen Röntgenbild schon ausgeprägte Osteophyten zirkumferent sichtbar, die das KI-berechnete Ergebnis unwahrscheinlich werden lassen. Dementsprechend muss man auch hier sorgfältig in der Strategieauswahl Augenmerk auf die Realisierbarkeit der Planung legen. Daneben konnte eine Arbeit zeigen, dass die reale postoperative Wirbelsäulenlordose nicht 1:1 der der Stabkontur entspricht, da auch die Schraubenlänge und Angulation in Relation zum Stab einen signifikanten Einfluss auf das Verhältnis von Stabbiegung zu Wirbelsäulenkrümmung haben [5]. Die mittlere Abweichung in der erwähnten Studie betrug 7,8° für die Lendenwirbelsäule und 9,1° allein für L4-S1 mit einem von Range von −24° bis 31°.

## Schlussfolgerung

Die operative Versorgung adulter Skoliosen stellt trotz Überlegenheit der klinischen Ergebnisse gegenüber der konservativen Therapie aufgrund der hohen Komplikationsraten eine Herausforderung dar. Moderne KI-Werkzeuge können helfen, den Planungsprozess zu vereinfachen und zu verbessern und über einen individualisiert vorgebogenen Stab den Plan in die Realität umzusetzen [29]. Dennoch ist aufgrund unberücksichtigter Parameter in der Datenbasis, der Freiheit der Operationsstrategiewahl und methodischer Einschränkungen noch keine 100 %ige Lösung, sondern allenfalls eine Reduktion des Problems zu erwarten. Unter steigendem Kostendruck ist die Zeit- und Aufwandsersparnis und die Reduktion der Komplikationsrate den zusätzlichen Kosten durch das KI-Tool/den individualisierten Stab gegenüberzustellen. Eine Kosten-Nutzen-Analyse steht derzeit jedoch noch aus. Bei Einbringen des heutigen Wissens in ein derartiges KI-Modell bietet sich jedoch eine große Chance auf eine verbesserte Versorgung der Patientenkohorte mit adulten Skoliosen.

## Fazit für die Praxis

- Adulte Skoliosen profitieren von der operativen Therapie, die Komplikationsrate ist jedoch hoch.
- Die Komplikationsrate ist abhängig vom individuellen Korrekturergebnis, aber auch von weiteren Faktoren.
- Künstliche Intelligenz für Planung und Stabkonturierung ist derzeit das beste Werkzeug ein individuell korrektes Alignment zu erreichen, unter Berücksichtigung von Osteoporose, Alter, Frailty und einer Realitätsprüfung der Planung (Kompensationsmöglichkeiten in Hüfte und Thorax, knöchernes Release in der Ausführung).
- Es ermöglicht eine schnelle Analyse, Planung und Kontrolle.
- Strategische Überlegungen des Chirurgen bleiben ein Risikofaktor für den Erfolg.
- Die Integration weiterer Risikofaktoren und von klinischen Outcome-Parametern bietet Optionen der Verbesserung.

## Data Availability

Im Rahmen dieser Studie wurden retrospektiv Patientendaten herangezogen und analysiert. Die Daten liegen anonymisiert den Autoren vor und sind in einer Datenbank gespeichert.

## Abkürzungen

GAP: Global Alignment and Proportion
KI: Künstliche Intelligenz
LIV: Unterer zu instrumentierender Wirbel
LL: Lumbale Lordose
NRS: Numerische Ratingskala
PI: „Pelvic incidence“
PI-LL: „Pelvic incidence“ lumbale Lordose Mismatch
PJF: Proximal junktionales Versagen
PLIF: Posteriore lumbale intersomatische Fusion
PSO: Pedikelsubtraktionsosteotomie
PT: „Pelvic tilt“
rhBMP: „Recombinant human bone morphogenetic proteins“
SPO: Smith-Peterson-Osteotomie
SVA: Sagittaler vertebraler Abstand
TK: Thorakale Kyphose
TLIF: Transforaminale lumbale intersomatische Fusion
UIV: Oberer zu instrumentierender Wirbel
